# Improved efficiency of *in situ* protein analysis by proximity ligation using UnFold probes

**DOI:** 10.1038/s41598-018-23582-1

**Published:** 2018-03-29

**Authors:** Axel Klaesson, Karin Grannas, Tonge Ebai, Johan Heldin, Björn Koos, Mattias Leino, Doroteya Raykova, Johan Oelrich, Linda Arngården, Ola Söderberg, Ulf Landegren

**Affiliations:** 1 Department of Pharmaceutical Biosciences, Pharmaceutical Cell Biology, Uppsala University, Uppsala, Sweden; 2 Department of Immunology, Genetics and Pathology, Science for Life Laboratory, Uppsala University, Uppsala, Sweden; 3 Department of Systemic Cell Biology, Max Planck Institute of Molecular Physiology, Dortmund, Germany

## Abstract

We have redesigned probes for *in situ* proximity ligation assay (PLA), resulting in more efficient localized detection of target proteins. *In situ* PLA depends on recognition of target proteins by pairs of antibody-oligonucleotide conjugates (PLA probes), which jointly give rise to DNA circles that template localized rolling circle amplification reactions. The requirement for dual recognition of the target proteins improves selectivity by ignoring any cross-reactivity not shared by the antibodies, and it allows detection of protein-protein interactions and post-translational modifications. We herein describe an improved design of the PLA probes –UnFold probes – where all elements required for formation of circular DNA strands are incorporated in the probes. Premature interactions between the UnFold probes are prevented by including an enzymatic “unfolding” step in the detection reactions. This allows DNA circles to form by pairs of reagents only after excess reagents have been removed. We demonstrate the performance of UnFold probes for detection of protein-protein interactions and post-translational modifications in fixed cells and tissues, revealing considerably more efficient signal generation. We also apply the UnFold probes to detect IL-6 in solution phase after capture on solid supports, demonstrating increased sensitivity over both normal sandwich enzyme-linked immunosorbent assays and conventional PLA assays.

## Introduction

It is well established that measurement of proteins in solution can be greatly improved if detection depends on dual recognition by antibodies in the form of sandwich immunoassays rather than binding by single antibodies^1^. Such assays are now routinely used for high-performance solution-phase protein detection in research and the clinic. By contrast, 75 years after the immunochemistry method was first described^2^, most *in situ* protein detection assays still rely on the selectivity of target binding by individual antibody preparations, often leading to unspecific detection of proteins other than the intended ones^3,4^. The *in situ* proximity ligation assay (PLA), first published a decade ago, represents an alternative strategy where *in situ* target detection depends on binding by pairs of oligonucleotide-conjugated antibodies, giving rise to circular DNA strands that are then amplified by rolling circle amplification (RCA)^5^. In this way, the assay achieves improved specificity by virtue of the dual recognition and enhanced signal strength by localized amplification via RCA^6^. *In situ* PLA has also become popular as a means to identify interacting proteins in cells and tissues^7–9^ or to apply pairs of antibodies in order to simultaneously detect both proteins and their post-translational modifications through *e*.*g*. phosphorylation, glycosylation or palmitoylation^10–12^. Localized detection of both individual proteins and signaling protein complexes serves to portray the heterogeneous nature of individual cells, a matter of particular relevance in malignancy with clonally distinct tumor cells intermixed with stromal tissue.

**Figure 1:**
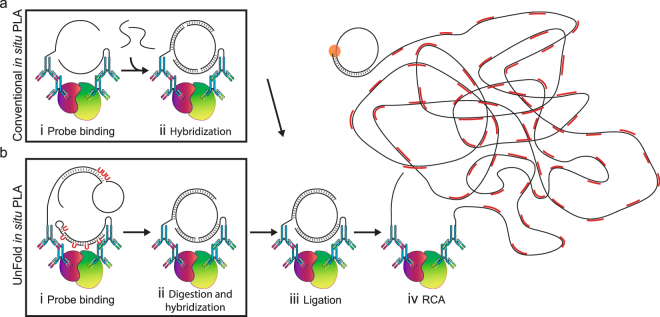
Schematic illustration of *in situ* PLA using conventional and UnFold probes. (**a**) Conventional *in situ* PLA. (**b**) *In situ* PLA using UnFold probes. (i) After pairs of primary antibodies have bound a pair of interacting proteins (red and green) followed by washes, secondary conventional or UnFold *in situ* PLA probes are added, followed after an incubation by renewed washes. (ii) In the conventional design under (**a**) two more oligonucleotides are then added that can form a DNA circle. Using the UnFold design in (**b**) the probe carrying a hairpin-loop oligonucleotide is cleaved at the U residues, liberating a free 5′ end capable of being ligated to the 3′ end of the same DNA strand. Meanwhile, the U residues in the hairpin DNA strand of the other UnFold probe are cleaved presenting a single-stranded template for the enzymatic joining of the ends of the strand on the first UnFold probe. (iii) A DNA ligase is added to form DNA circles in the two variants of *in situ* PLA. (iv) Finally, phi29 DNA polymerase is added to initiate RCA primed by oligonucleotides on one of the antibodies, and fluorescent oligonucleotides are used to visualize the RCA products.

*In situ* PLA uses oligonucleotide-modified antibodies, referred to as PLA probes or proximity probes, to visualize target proteins. Upon proximal binding by pairs of PLA probes, the conjugated oligonucleotides template ligation of secondarily added oligonucleotide pairs to generate DNA circles. Replication of the DNA circles through RCA is then primed by one of the PLA probes, resulting in prominent signals at the sites of antibody binding (Fig. 1a). Each RCA product consists of a single DNA strand with several hundred complements of the DNA circle, collapsed into a micrometer-sized DNA bundle that is suitable for detection and digital enumeration by microscopy after hybridization with fluorophore- or enzyme-labeled detection oligonucleotides^13^.

*In situ* PLA has also been applied to improve sensitivity, specificity, and target range in other methods for localized protein detection, for example, western blotting^14^, flow cytometry^15,16^, and sandwich enzyme-linked immunosorbent assay (ELISA)^17,18^.

We describe herein a modified design for PLA probes in the form of so-called UnFold probes that incorporate all elements required for the production of the circular amplification templates. We evaluate this new design in several different applications and demonstrate improved efficiency of detection compared to conventional *in situ* PLA, both *in situ* and in microtiter wells, with preserved signal-to-noise ratios.

## Results

### UnFold probe design

In the UnFold design of probes for *in situ* PLA, one of the antibodies carries a circle-forming oligonucleotide, while the other antibody is conjugated to an oligonucleotide that can template the ligation reaction required to create this DNA circle, avoiding the need for adding separate oligonucleotides (Fig. 1). The circle-forming oligonucleotide (circle probe) has a hairpin-loop structure with cleavable DNA uracil (U) residues to release the 5′ end of the loop, while the template probe contains a hairpin with several U residues in the distal 3′ portion of a DNA hairpin attached to an antibody (Fig. 1b). When the two DNA-conjugated antibody probes are first added to a sample, their DNA strands are unable to interact, since the hairpin in the template probe shields the segment complementary to the circle probe. Each antibody, therefore, must recognize its target independently to remain bound after washes^19^. After unbound probes have been removed, a subsequent enzymatic “unfolding” step allows the pairs of probes to form DNA circles by templated ligation. The unfolding step is achieved by removing U bases in the oligonucleotides using the enzyme uracil-DNA glycosylase (UNG), and then cleaving the sugar-phosphate backbone of the abasic residues using endonuclease IV (EndoIV). For the circle probe, this has the effect of liberating the 5′ end of the circle-forming DNA strand, which remains hybridized to a DNA strand covalently attached to its antibody. Meanwhile, the 5′ segment of the hairpin-modified template probe is now available for hybridization to the free 5′ and 3′ ends of the circle forming DNA strand, since the complementary 3′ segment containing U residues has been degraded. Thereby the ends of the circle-forming DNA strands are joined by a ligase, giving rise to circular DNA strands that remain hybridized to the PLA probes (Fig. 1ii). The addition of phi29 DNA polymerase then executes RCA, primed by the PLA probes, and the localized RCA products can be visualized using fluorophore-labeled detection oligonucleotides.

### Comparison of conventional *in situ* PLA probes and UnFold probes for analysis of cells and tissue sections

Secondary antibodies were conjugated to oligonucleotides and then purified and validated to create the conventional and UnFold *in situ* PLA probes (Supplementary Figs S1 and S2). To evaluate the UnFold probes and compare them to the conventional *in situ* PLA probes, we used previously validated combinations of primary antibodies targeting the adherens junction protein E-cadherin with its interaction partner β-catenin^20^, and phosphorylated forms of the PDGF receptor β (PDGFR-β)^10^.

**Figure 2:**
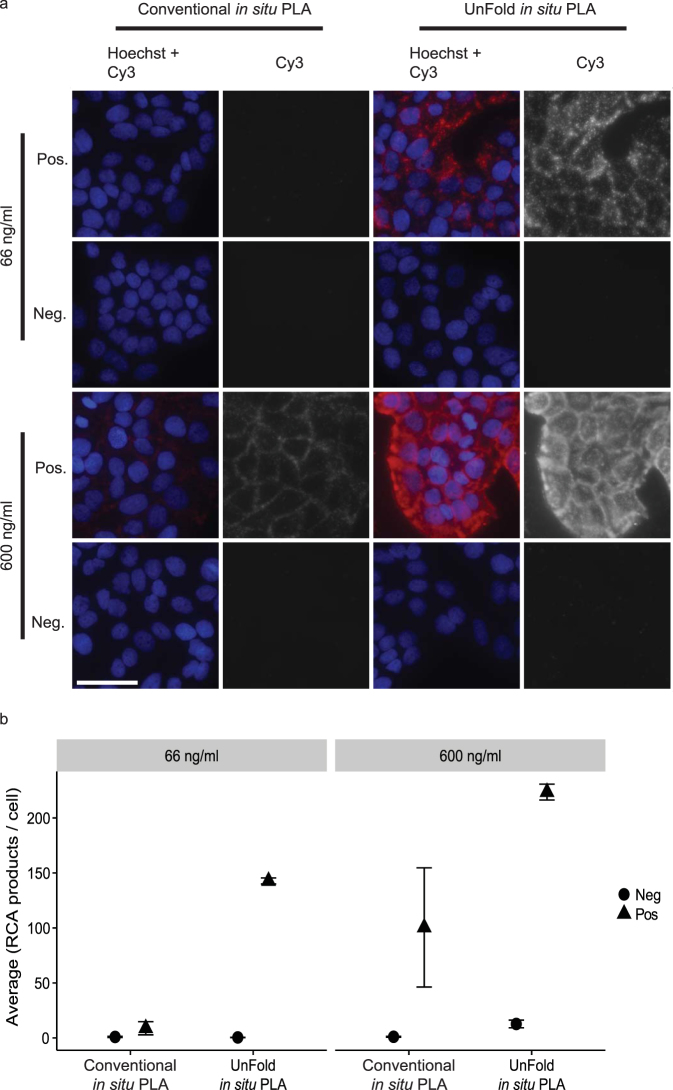
Visualization of E-cadherin/β-catenin interactions in HaCat cells. (**a**) Images of cells stained with primary antibodies against E-cadherin and β-catenin and secondary conventional or UnFold *in situ* PLA probes recording E-cadherin/β-catenin interactions (Pos). As a background control, primary antibodies were omitted (Neg). The RCA products were labeled with Cy3 (red dots in the merged images) and the nuclei were stained with Hoechst 33342. Scale bar (white) = 50 µm. (**b**) Quantification of signals per cell comparing *in situ* PLA and UnFold at different probe concentrations. Experiments were performed three times. Three images per experimental condition were acquired. Error bars represent SEM.

**Figure 3:**
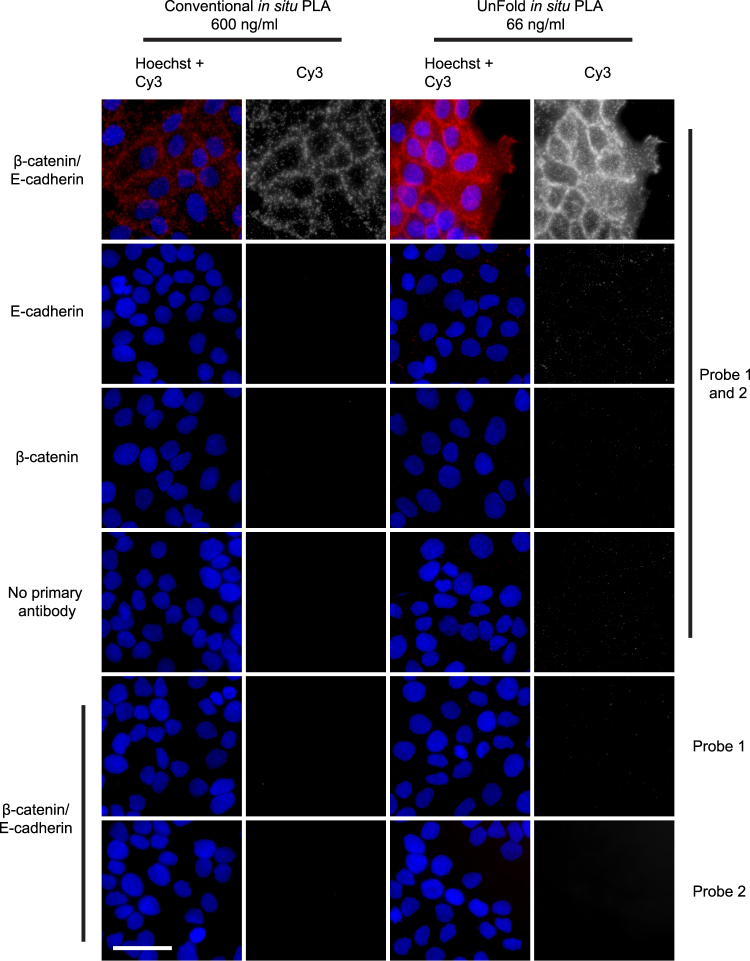
Image panel of HaCat cells showing detection of β-catenin/ E-cadherin interactions along with several technical controls. In the top row images, both of the primary antibodies and the PLA probes were included, revealing the characteristic β-catenin/ E-cadherin interaction stain. For the remaining rows in the panel, one or more of the primary or secondary antibodies were excluded. All signals were stained with Cy3 (Red in merge images and in grayscale) and the nuclei were stained with Hoechst 33342 (Blue). Scale bar (white) = 50 μm.

**Figure 4:**
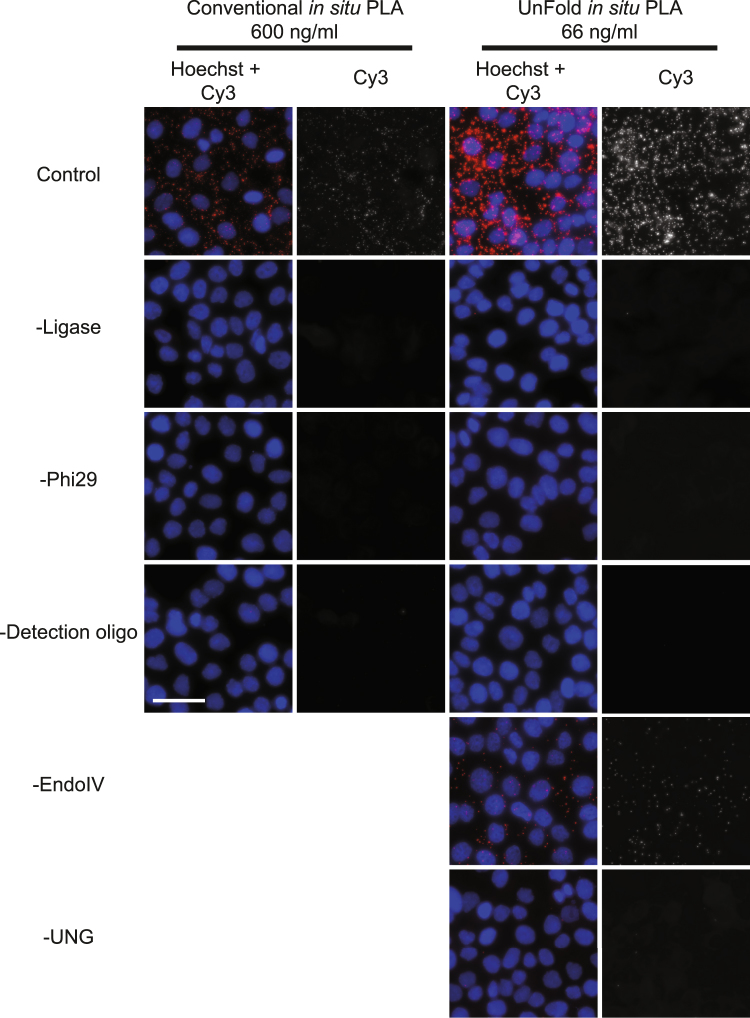
Image panel of HaCat cells showing detection of β-catenin/E-cadherin interaction or different technical controls, omitting enzymes. For the top image row, both of the primary antibodies and the PLA probes were included, revealing the characteristic staining pattern of β-catenin/E-cadherin interactions. The remaining rows in the panel show the effect of removing enzymes or detection oligonucleotides as indicated in the figure. All signals were stained with Cy3 (Red in merge images or in grayscale) and the nuclei were stained with Hoechst 33342 (Blue). Scale bar (white) = 50 μm.

We used a dilution series of DNA-conjugated antibodies for the two probe designs to achieve optimal signal-to-noise ratios (Supplementary Fig. S3). Signals representing E-cadherin/β-catenin interactions in HaCat cells were compared to background signals when no primary antibodies had been added. For conventional *in situ* PLA, we tested the probes at 66, 200, 600 and 1800 ng/ml. Based on this analysis we selected 600 ng/ml of the antibody-DNA conjugates to investigate receptor signaling in cells, and 1800 ng/ml in tissue sections, which commonly require a higher probe concentration. For UnFold probes, the concentrations tested were 20, 66, 200 and 600 ng/ml and the optimal signal-to-noise was obtained at 66 ng/ml. At higher probe concentrations, the RCA products become so abundant that they start to coalesce and can no longer be counted as individual signals. This results in an apparent reduction of signals, while the amount of unspecific background signals is increased (Fig. 2). The effect is a lower signal-to-noise ratio at higher concentrations, as also observed in conventional *in situ* PLA^21^. We also investigated unspecific signals by omitting either of the primary antibodies or by excluding the PLA probes (Fig. 3). When omitting one of the primary antibodies the amount of signals from cross-reactivity and/or nonspecific binding was low. Without the presence of both PLA probes, minimal staining was detected. Omitting any of the enzymes removed all signals from the cells, except in the case of endoIV, in which case some specific signals were still observed in its absence (Fig. 4). The treatment with UNG removes uracil bases and creates abasic sites. The abasic sites are fragile and prone to spontaneous breakage also in the absence of endoIV^22^. Nonetheless, addition of endoIV dramatically increases the strand breaks necessary for production of circular RCA templates and contributes to efficient signal generation.

**Figure 5:**
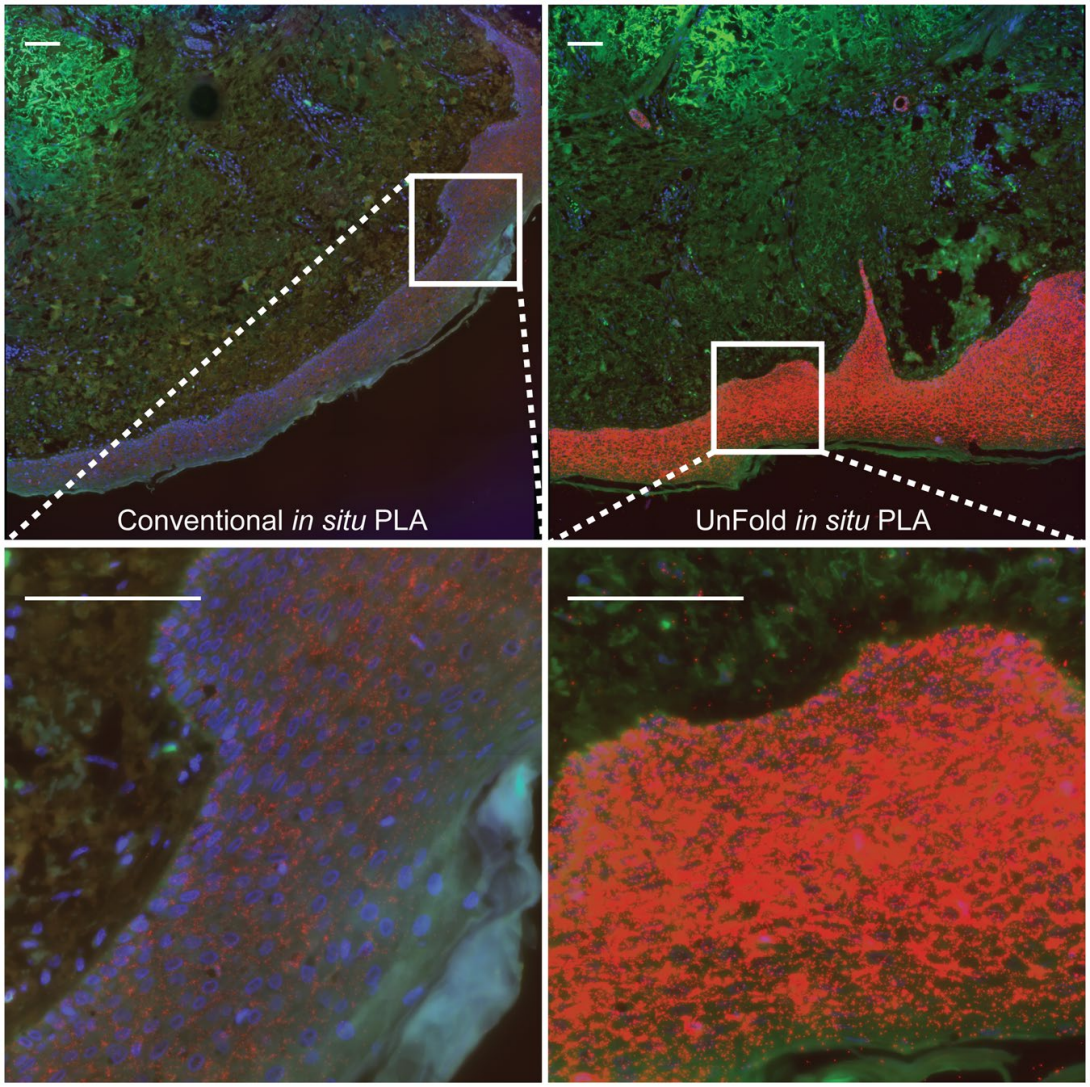
Visualization of the interaction of β-catenin and E-cadherin in skin tissue. This stitched tile scan of stained skin tissue shows the specificity of the β-catenin and E-cadherin interaction (red) in the epidermis. The autofluorescence in the FITC channel (green), together with the nuclear Hoechst staining (blue) allows visualization of the different structures in the tissues. Each tissue section was stained with conventional or UnFold *in situ* PLA probes at a concentration of 1800 ng/ml and 66 ng/ml, respectively. Scale bar (white) = 100 µm. This experiment was performed once.

To evaluate the performance of UnFold probes in formalin-fixed paraffin-embedded (FFPE) material, we analyzed interactions between E-cadherin and β-catenin in skin tissue sections. E-cadherin is known to be prevalent in epithelial cells that are present in the epidermis but absent in the dermis^23^. After trying different concentrations of the antibody-DNA conjugate, we found that 1800 ng/ml for conventional *in situ* PLA and 66 ng/ml for UnFold probes resulted in signals specifically clustering in the epidermis (Fig. 5). The number of signals in the epidermis was greater for UnFold, compared to conventional *in situ* PLA, even though only 1/27 of conjugate concentration was used.

**Figure 6:**
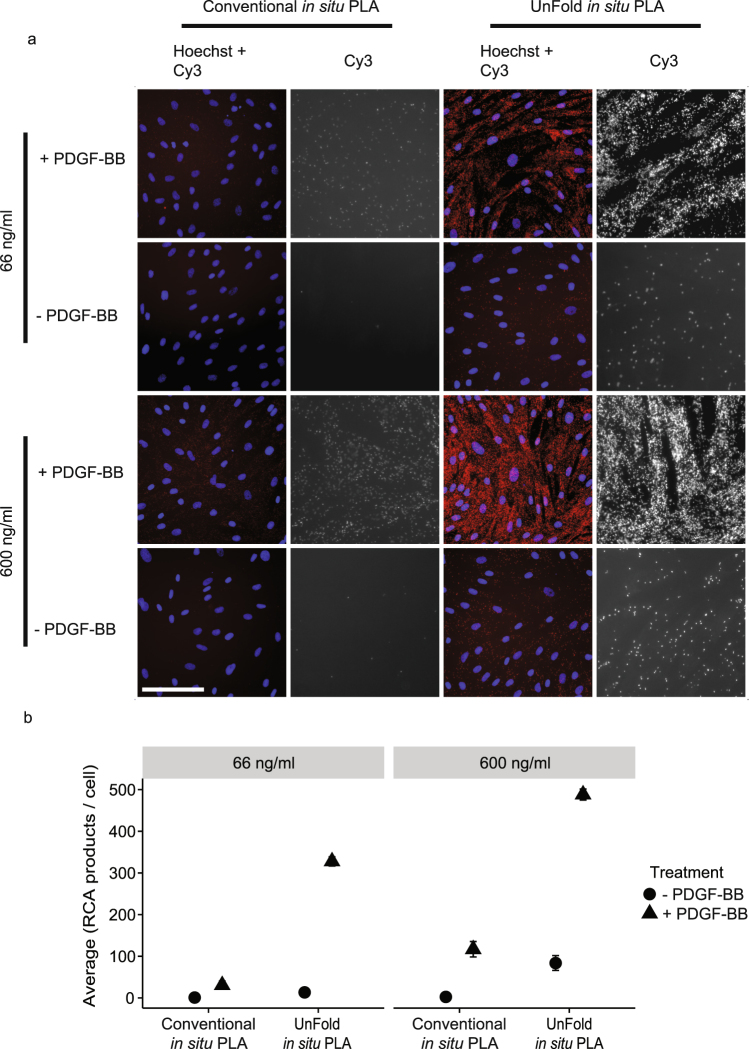
Visualization of phosphorylation of PDGFR-β in BJ hTert cells. (**a**) Images comparing serum-starved BJ hTert cells (−) versus cells treated with PDGF-BB for 45 min on ice (+) by detecting phosphorylation levels of the PDGFR-β (anti-PDGFR and anti-pan phosphorylation (pY100) antibodies). The RCA products were labeled with Cy3 (red dots in the merged images) and the nuclei were stained with Hoechst 33342. Scale bar (white) = 50 µm. (**b**) Quantification of signals comparing conventional and UnFold *in situ* PLA at different probe concentrations. The experiment was performed four times. Five images were collected per experimental condition. Error bars represent SEM.

We next compared the efficiency of detecting phosphorylated proteins by using either conventional *in situ* PLA probes or UnFold probes. Immortalized fibroblasts, BJ hTert cells, were starved or stimulated with platelet-derived growth factor-BB (PDGF-BB). Both designs for pairs of secondary antibody-oligonucleotide conjugates detected increased phosphorylation of the PDGFR-β (Fig. 6a) in stimulated cells using anti-PDGFR and anti-pan phosphorylation (pY100) as primary antibodies. UnFold probes revealed a greater number of phosphorylation events than conventional *in situ* PLA at both probe concentrations tested (66 and 600 ng/ml, Fig. 6b). Signals at the higher probe concentrations coalesced and hence individual RCA products could not be accurately resolved, preventing digital counting of the signals^21^. UnFold probes (66 ng/ml) were also more efficient than conventional *in situ* PLA reagents (1800 ng/ml) in detecting phosphorylation of AKT and ERK1/2 in response to stimulation with PDGF, downstream in the signaling cascade (Supplementary Fig. S4). The protein phosphorylations described above were validated by Western blot (Supplementary Figs S5 and S6). In addition to these assays, the specific phosphorylation of tyrosine 1068 (pY1068) of the epidermal growth factor receptor (EGFR) was quantified upon stimulation with EGF in HCT116 colorectal carcinoma cells (Supplementary Fig. S7). In line with results from the previous experiments, UnFold probes (66 ng/ml) yielded an about four fold higher ratio of signals comparing EGF-stimulated versus unstimulated cells than the conventional *in situ* PLA. The phosphorylation of tyrosine residue 542 in Src homology region 2 domain-containing phosphatase-2 (Shp2) was measured in the same cell line (Supplementary Fig. S7). Here, the results show a clear difference of the UnFold design in terms of signal in comparison to the conventional *in situ* PLA.

### Analysis of proteins in solution phase

PLA has previously been shown to increase the sensitivity of detection in sandwich assays for protein detection in solution phase, where one antibody is used for target capture from liquid samples, followed after washes by detection with two DNA-conjugated antibodies that undergo PLA reactions^24,25^. These assays allow interrogation of larger sample volumes, containing proportionally more target molecules, and they allow washes to remove excess reagents. This is in contrast to homogenous PLA or proximity extension reactions (PEA) that depend on small reaction volumes and background reduction by dilution but no washes before ligation or polymerization. Moreover, the requirement for target binding by sets of three rather than two antibodies to generate an amplifiable DNA signal reduces the risk for cross-reactive detection of irrelevant proteins^26,27^. The DNA products that form may be amplified by PCR or alternatively, using the *in situ* PLA mechanism, by RCA for isothermal amplification^17,18^.

**Figure 7:**
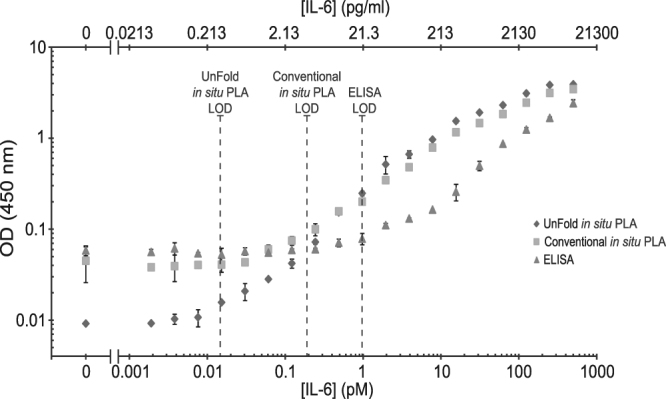
Detection of recombinant IL-6 from solution phase using UnFold probes. Microtiter wells were precoated with capture antibodies, which served to bind the antigen, IL-6. Captured target proteins were then detected using a standard sandwich enzyme-linked immunosorbent assay (ELISA), or via conventional or UnFold *in situ* PLA probes. In each case, the signals were recorded by an HRP-mediated colorimetric reaction. After addition of the HRP substrate, TMB, the absorbance was recorded by spectrophotometry. The experiment was performed four times and one representative graph is shown. Error bars = Standard deviation of duplicates, dashed lines indicate the experimental LODs.

The increased efficiency of signal generation that we observed in cells and tissues using UnFold probes compared to conventional *in situ* PLA reagents motivated a comparison of the two approaches also for detection of proteins in solution after capturing on solid supports. We immobilized antibodies in microtiter wells to capture IL-6, and the trapped antigens were then detected by an enzyme-conjugated antibody reagent for regular ELISA, or via *in situ* PLA using pairs of either conventional or UnFold probes. In line with our *in situ* studies, we found that the UnFold design achieved a lower limit of detection (LOD) compared to the conventional *in situ* PLA design, which in turn exhibited improved performance over ELISA (Table 2, Fig. 7). Further experiments where antigen was diluted in 10% or 100% cell lysates, similarly demonstrated the improved performance of the UnFold probes over conventional *in situ* PLA probes and sandwich ELISA (Supplementary Fig. S8).

**Table 2:** Analytical characteristics.

IL-6	UnFold *in situ* PLA	Conventional *in situ* PLA	ELISA
LOD_mean_ (pM)	0.022	0.082	1.803
LLOQ_mean_ (pM)	0.104	0.465	5.861
R^2^_mean_	0.996	0.996	0.997
Intra assay variation_mean_ (%)	11.2	11.7	9.49
Inter assay variation_mean_ (%)	30.0	11.6	10.6

All values are averages of four separate experiments.

## Discussion

Immunohistochemistry (IHC) is an established technique in research and routine pathology. A growing interest in imaging the distribution of single or large sets of proteins in cells and tissues is still mostly served by variants of the IHC or immunofluorescence techniques where binding by single labeled antibodies is monitored. With this publication, there are now three alternative technologies where target binding by pairs of antibodies is required to elicit a signal via localized amplification reactions. The *in situ* PLA technique first presented in 2006^5^, the proximity hybridization chain reaction (ProxHCR)^28^, and with this paper now also UnFold *in situ* PLA reactions. All these three techniques share the advantage that cross-reactive detection of irrelevant proteins is less likely since only targets recognized by pairs of antibodies can give rise to detection signals, greatly reducing risks of nonspecific background^29,30^. The three techniques are also all useful to investigate interactions or close proximity between pairs of proteins where each protein is bound by one antibody, and the same principle also holds for detection of posttranslational modifications, where one antibody is devoted to detection of the protein and another recognizes the modification, as illustrated herein for protein phosphorylations. Among the three, the ProxHCR reaction has the advantage over the other two dual-binding assays that no enzymes are required, which reduces cost and simplifies automation using staining workstations due to a simple assay format. However, staining tends to be weaker than for *in situ* PLA, and signals do not lend themselves for digital enumeration because of the more even distribution that is more similar to regular IHC or immunofluorescence. The UnFold technique differs from the earlier version of the *in situ* PLA technique in that all DNA elements required for the amplified detection are included in the reagents. The oligonucleotides conjugated to pairs of antibodies are prevented from hybridizing to each other prematurely by hairpin structures, which are removed only after antibodies have independently bound their protein targets, followed by washes. After excess reagents have been removed by washes, the UnFold probes are treated (unfolded) so that an oligonucleotide on one of the antibodies can template circularization of an oligonucleotide hybridized to an oligonucleotide conjugated to another antibody, having bound in proximity. Compared to the conventional *in situ* PLA probe design we find that UnFold *in situ* PLA probes improve the efficiency of signal generation for *in situ* PLA as an effect of the inclusion of the circularization oligonucleotide on one of PLA probes, and the single ligation reaction required to form DNA circles. This serves to lower the risk of formation of linear ligation products that would fail to be detected rather than the desired DNA circles capable of templating RCA.

*In situ* PLA reactions can be performed in multiplex^8^, and the more efficient UnFold design should also lend itself for the construction of multiplex detection reactions, a matter of increasing importance for analyses of cells and tissues. A number of approaches are available to read out multiplex molecular detection reactions by microscopy *in situ* or flow cytometry *in situ*, through methods such as sequential bleaching^31^, *in situ* sequencing^32,33^, sequential hybridization^34^ or, by mass cytometry using lanthanide labeled probes^34–36^.

The improved efficiency of detection using UnFold probes demonstrated here means that also weakly expressed proteins can be detected. If a digital readout is desired for abundant proteins then lower concentrations of the UnFold probes may be used. Alternatively, the assay can be modified so that only a given fraction of all RCA products are detected as previously described^21^. It is also possible to use shorter incubations for the RCA reaction to achieve a more homogenous staining to be evaluated according to its intensity rather than by counting individual reaction products.

In conclusion, UnFold probes improve the efficiency of *in situ* PLA and they can thereby enhance the sensitivity of detection of immobilized target proteins *in situ* and in microtiter wells.

## Materials and Methods

### Cell culture

The keratinocyte cell line HaCaT and the immortalized fibroblast cell line BJ hTert, both of human origin, were grown at 37 °C and 5% CO_2_ in Dulbecco’s modified Eagle’s medium (DMEM) supplemented with 10% fetal bovine serum (FBS), 50 units/ml penicillin, 50 μg/ml streptomycin and 1 mM L-glutamine (all from Thermo Scientific).

### Oligonucleotide design

Sequences with suitable characteristics were designed and evaluated *in silico* using the NUPACK software package (www.nupack.org)^37^. All aldehyde-modified oligonucleotides were purchased from Trilink Biotechnologies and other oligonucleotides were acquired from Integrated DNA Technologies (Table 1).

**Table 1:** Oligonucleotide sequences.

Design	Oligonucleotides	Sequence
Conventional *in situ* PLA	Probe 1	5′ aldehyde or azide – AAAAAAAAAATATGACAGAACTAGACACTCTT
	Probe 2	5′ aldehyde or azide – AAAAAAAAAAGACGCTAATAGTTAAGACGCTT – 3 × 2′ O-methyl RNA uracil
	Circularization oligonucleotide 1	5′ phosphate – GTTCTGTCATATTTAAGCGTCTTAA
	Circularization oligonucleotide 2	5′ phosphate – CTATTAGCGTCCAGTGAATGCGAGTCCGTCTAAGAGAGTAGTAC-AGCAGCCGTCAAGAGTGTCTA
UnFold *in situ* PLA	Probe 1	5′ aldehyde or azide – AAAAATATGACAGAACTAGACACTCUUUCTATTAGCGTCCAGTGA-ATGCGAGTCCGTCTGAAAGAGTGTCTAGTTCTGTCATATTTAAGCGTCTTAA
	Probe 2	5′ aldehyde or azide – AAAAAAGACGCTAATAGTTAAGACGCTTUUUAAAAAAAAGCGUC-UUAACUAUUAGCGUC
All	Detection oligonucleotide	5′ Cy3 or HRP– CAGTGAATGCGAGTCCGTCT – 3 × 2′ O-methyl RNA uracil

### Conjugation of secondary antibodies

Donkey anti-rabbit, donkey anti-mouse and donkey anti-goat antibodies (Jackson ImmunoResearch), 350 µg each, were concentrated using the Amicon Ultra 10 K centrifugal filter unit (Merck Millipore) according to the manufacturer’s instructions to a concentration >3 mg/ml in PBS. S-HyNic Crosslinker (Solulink) was dissolved in dimethyl sulfoxide (DMSO) (Sigma Aldrich) to 20 mM and the crosslinker and antibodies were mixed with a 25-fold molar excess of crosslinker over antibodies. The mix was incubated protected from light with gentle rotation at room temperature for 2.5 hours. After activation of the antibodies, the buffer was exchanged for 150 mM NaCl and 100 mM NaHPO_4_ pH 6.0 using prewashed Zeba Spin Desalting Columns 7 K MWCO (Thermo Scientific). The antibodies were then mixed with aldehyde-modified oligonucleotides (Table 1) at an antibody:oligonucleotide molar ratio of 1:3. Aniline was added to a final concentration of 10 mM to catalyze the conjugation reaction. The antibody-oligonucleotide mix was incubated protected from light with gentle rotation at room temperature for 2 hours. Immediately after the incubation, the buffer was exchanged to PBS using a prewashed Zeba Spin Desalting Column 7 K MWCO. The conjugated antibodies were purified from remaining unconjugated antibodies and oligonucleotides by ÄKTA Pure HPLC (GE Healthcare) using Superdex 200 10/300 column (GE Healthcare). The collected fractions from the HPLC purification were validated by electrophoresis. Briefly, the conjugates were mixed with Novex TBE-Urea Sample buffer (Life Technologies) and separated on a Novex TBE-Urea Gel 10%, 15 wells (Life Technologies) at 180 V for 50 min. DNA was visualized using SYBR Gold Nucleic Acid Gel Stain (Life Technologies) and protein using Coomassie stain (Bio-Rad, Hercules, USA). The SYBR Gold staining was visualized with Bio-Rad Gel-Doc XR (Bio-Rad) and the Coomassie staining using an Odyssey scanner (Li-Cor Bioscience) at a wavelength of 700 nm. Concentrations of the conjugates were determined using the Dot-it Spot-it Total Protein Assay (http://dot-it-spot-it.com, Maple Stone AB)^38^ according to the manufacturer’s instructions with the sample diluted in PBS containing 1% SDS and a known AB reference donkey anti-rabbit/ donkey anti-mouse dilution series for comparison. Dot-it Spot-it membranes were scanned using Epson Perfection V600 Photo (Epson) and analyzed using ImageJ and Excel. About 1–15% of the secondary antibody is recovered as conjugated probes.

### Conjugation of primary antibodies

Polyclonal anti-human IL**-**6 antibodies (AF-206-NA, R&D Systems) were covalently coupled to oligonucleotides. 10 µg of antibodies per conjugated PLA probe at a concentration of 2 µg/µl in PBS was activated by addition of a 33.3-fold molar excess of dibenzyl cyclooctyne NHS ester (DBCO-NHS ester; Jena Bioscience), freshly dissolved in DMSO (Sigma-Aldrich) and incubated at room temperature for 30 min. Thereafter, the activated antibodies were purified from DBCO-NHS using the 7 K MWCO Zeba Spin columns (Thermo Scientific) that had been equilibrated with PBS. The activated antibodies were then split into two aliqotes, mixed with a 2.5-fold molar excess of the respective azide-modified oligonucleotides, and incubated overnight at 4 °C.

### Application of probes *in situ*

HaCat cells were seeded at a density of approximately 120 000 cells/cm^2^ and grown for 1–2 days in 8-well Lab-Tek II Chamber Slides (Sigma-Aldrich) until 70–80% confluent. Approximately 47 000 cells/cm^2^ of BJ hTert cells were seeded in chamber slides and grown overnight. The BJ hTert cells were serum-starved in DMEM overnight. The cells were subsequently incubated with or without 50 ng/ml PDGF-BB in DMEM for 1 hour on ice. Alternatively, to investigate phosphorylation of downstream signaling proteins the cells were incubated for 15 min at 37 °C. The cells were washed twice in ice-cold PBS before fixation with 3.7% formaldehyde on ice for 15 min, followed by two consecutive washes in PBS and dehydration in 70% EtOH before being stored at −20 °C until use.

Approximately 15 000 HCT116 cells per well were seeded in a 96 well plate (NUNC) and left to adhere for 48 hours. Subsequently, cells were serum starved for 5 hours in DMEM. Then the cells were incubated with 100 ng/ml EGF for 3 min at 37 °C. Directly after stimulation cells were fixed with 3.7% formaldehyde on ice for 30 min, followed by two consecutive washes with PBS. Cells were used directly.

Anonymized FFPE skin sections on glass slides were provided by Olink Bioscience AB and their use has been approved by the local ethical standards committee (Uppsala 2005:347). Tissue sections were treated according to standard methods. The slides were deparaffinized in 100% xylene (three consecutive incubations for 5 min, 5 min and 1 min). Next, tissues were rehydrated in decreasing ethanol concentrations, starting from 99% ethanol twice for 3 min, 95% ethanol twice for 5 and 3 min, respectively, and finally, 70% ethanol for 3 min. The slides were subsequently washed in water three times and subjected to antigen retrieval. The latter was performed in a pressure cooker, where slides were boiled in 1 × Dako Target retrieval solution (Agilent) at 2 atm pressure and 125 °C for 4 min and then at 90 °C for 3 min. The tissue sections were then allowed to gradually cool down to room temperature and washed in water. Thereafter, the samples were stored in PBS until use.

The thawed cells were rehydrated in PBS for 5 min at room temperature and permeabilized with 0.2% TritonX100 in PBS for 5 min at room temperature, followed by washes in PBS. For the EGFR and Shp2 assays, the cells were permeabilized with 0.1% TritonX100 in PBS for 5 min at room temperature and then the phospho-sites were made available by incubation with 1% SDS in PBS for 5 min, followed by extensive washes with PBS.

The cells and tissue sections were incubated with 50% Odyssey blocking buffer (Li-Cor Bioscience) in 1 × Tris-buffered saline (TBS) for 30 min at 37 °C. Rabbit anti-β-catenin antibodies (#7199, Santa Cruz Biotechnology) were applied at a dilution of 1:200 diluted in blocking buffer, mouse anti-E-cadherin antibodies (#610182, BD biosciences) at 1:100, rabbit anti-PDGFR-β antibodies (#3169, Cell Signaling Technology) at 1:100, mouse anti-pY100 antibodies (#9411, Cell Signaling Technology) at 1:200, mouse anti-Akt antibodies (#2920, Cell Signaling Technology) at 1:100, rabbit anti-pAkt antibodies (#4060, Cell Signaling Technology) at 1:50, mouse anti- ERK1/2antibodies (#4696, Cell Signaling Technology) at 1:100 and rabbit anti-p ERK1/2antibodies (#4370, Cell Signaling Technology) at 1:100. We used goat anti-EGFR antibodies (AF231, RnD Systems) at 1:10 000, rabbit antibodies directed against pY1068 EGFR (#3777, Cell Signaling Technology) at 1:1000, goat anti Shp2 (PA5–17956, Thermo Fisher) at 1:1000, and rabbit anti pY542 Shp2 (ab62322, Abcam) at 1:5000. Paired combinations of rabbit and mouse, and goat and rabbit primary antibodies were incubated with samples overnight at 4 °C in a humidified chamber. After incubation, the slides were washed 3 times for 3 min each in TBS with 0.05% Tween 20 (TBS-T), before addition of secondary probes.

Conventional probes for *in situ* PLA or UnFold probes were diluted in blocking buffer to 20, 66, 200, 600 or 1800 ng/ml, and incubated on the slides for 60 min at 37 °C. After incubation, the slides were washed 3 times for 3 min each in TBS-T.

UnFold probes were digested by addition of 0.05 U/µl UNG and 0.025 U/µl endoIV in digestion buffer (20 mM Tris-HCl (pH 7.6), 30 mM NaCl, 1 mM EDTA, 100 mM KCl and 1 nM dithiothreitol (DTT)) supplemented with 0.25 mg/ml BSA (Sigma-Aldrich) for 45 min at 37 °C. The slides were then washed twice in TBS-T for 3 min. Circularization oligonucleotides for the two probe designs were ligated using 0.02 U/µl T4 DNA ligase (Thermo Scientific) in T4 DNA ligase buffer supplemented with 0.25 mg/ml BSA for 30 min at 37 °C, thereafter the slides were washed twice for 3 min in TBS-T. RCA was performed by addition of 0.5 U/µl phi29 polymerase (Thermo scientific) in phi29 polymerase buffer (Thermo Scientific) supplemented with 7.5 ng/ml PolyA (Sigma-Aldrich), 0.25 mM dNTP and 0.25 mg/ml BSA for 60 min at 37 °C, followed by two 3 min washes in TBS-T. The RCA products and the nuclei were visualized by incubating the slides with 0.025 µM fluorescence-labeled detection oligonucleotide and 40 µg/ml Hoechst 33342 in PBS supplemented with 2.5 µg/ml salmon sperm DNA and 0.25 mg/ml BSA for 30 min at 37 °C. Prior to mounting with Vectashield mounting medium (Vector Laboratories), the slides were washed twice for 10 min in 1xTBS and once for 15 min in 0.2 × TBS.

### Western blot

The stimulations were done essentially as described in Heldin *et al*., 2017^39^. Briefly, BJ hTert cells were transferred to a cell culture plate (50 000 cells/cm^2^) and let to adhere and spread overnight, thereafter the cells were starved overnight and stimulated with 50 ng/ml PDGF-BB at 37 °C or on ice as indicated. The stimulation on ice was preceded by a 10 min preincubation on ice. Following the specified treatments, cells were briefly washed in PBS on ice and lysed in 2 × NuPAGE™ LDS Sample Buffer supplemented with 100 mM DTT (hereafter referred to as sample buffer). Samples were denatured at 95 °C for 5 min before being subjected to a Bis-Tris polyacrylamide gel electrophoresis and subsequently transferred to a PVDF membrane using the Iblot2. Chameleon Duo (Li-core) was used as a molecular size marker. Membranes were blocked with Odyssey blocking buffer (LI-COR Biosciences, diluted 1:3 in TBS) for 2 hours before being incubated overnight at 4 °C with primary antibodies (rabbit anti-PDGFR-β (#3169, Cell Signaling Technology) at 1:1000, mouse anti-pY751- PDGFR-β (#3166, Cell Signaling Technology) at 1:1000, Rabbit anti-pY857- PDGFR-β (#3170, Cell Signaling Technology) at 1:1000, mouse anti-AKT (#2920, Cell Signaling Technology) at 1:500, rabbit anti-pAKT (#4060, Cell Signaling Technology) at 1:500, β-actin (sc-47778, Santa Cruz Biotechnology) at 1:1000 and rabbit anti-p ERK1/2 (#9101, Cell Signaling Technology) at 1:1000). Membranes were washed 4 × 10 min in 0.05% Tween-20 in TBS and incubated with the appropriate fluorescently tagged secondary antibodies (Alexa 680 and IRDye800) diluted in blocking buffer. The membranes were finally washed in 0.05% Tween-20 in TBS for 4 × 10 min and finally with TBS for 10 min. All membranes were scanned using an Odyssey Scanner (LI-COR Biosciences).

### Unfold versus conventional *in situ* PLA probes compared to ELISA for solution-phase protein detection

Wells in a 96-well microtiter plate (R&D Systems) were coated with 800 ng per well of human IL-6 antibodies (AF-206-NA, R&D Systems), diluted in coating buffer (1.5 g Na_2_CO_3_, 2.93 g NaHCO_3_, pH 9.6) at 4 °C overnight. Thereafter, the wells were blocked with 1% BSA in 1× PBS for 60 min at RT. The plates were then washed in washing buffer (1× PBS, 0.5% BSA and 0.05% Tween 20), and the same washes were used after each step in the assay.

Recombinant human IL-6 protein (206-IL-010, R&D Systems) was diluted in PLA buffer to concentrations ranging from 500 pM to 0 pM to make the standard curve and was added to the wells, followed by washes. The conventional *in situ* PLA probes and UnFold probes were incubated in PLA buffer (0.1% BSA (New England Bio-labs), 0.05% Tween (Sigma-Aldrich), 0.1 µg/µl salmon sperm DNA (Invitrogen), 100 nM goat IgG (Sigma-Aldrich), 1 mM D-biotin (Invitrogen), 5 mM EDTA, 1× PBS) for 5 to 10 min to block unspecific binding before combining the pairs of probes.

Conventional *in situ* PLA or UnFold probes at a final concentration of 150 ng/ml were added to each well, followed by incubation at room temperature for 60 min. After two washes, 50 µl of 0.05 U/µl UNG, 0.01 U/µl EndoIV in 1× EndoIV buffer (NEB) was added in reactions involving UnFold probes, followed by incubation for 30 min at 37 °C and then washes.

Thereafter, a ligation mix (0.25 mg/ml BSA, 1× T4 DNA ligase buffer without DTT, 1 mM ATP, 2.5 mM NaCl, 125 nM of the two circularization oligonucleotides (for conventional *in situ* PLA), 0.02 U/μl T4 DNA ligase (New England Bio Labs),) were added to each well and incubated for 30 min at 37 °C, followed by washes. Afterwards, the RCA mix (0.25 mg/ml BSA, 1× Phi29 polymerase buffer (Fermentas), 0.25 mM dNTPs (Fermentas), 0.05 U/µl Phi29 DNA polymerase (Fermentas) was added to each well, followed by incubation at 37 °C for 90 min and subsequent washes. A hybridization solution containing 50 pM horseradish peroxidase-labeled detection oligonucleotides in 20% formamide and 2× SSC was added to the wells and incubated for 30 min at 37 °C, followed by washes. Finally, 50 μl of the horseradish peroxidase-substrate tetramethyl benzidine (TMB; Sigma-Aldrich), equilibrated at RT, was added to each well and incubated for 10 min. 50 µl of 1 M H_2_SO_4_ was added to each well to stop the reaction and the colorimetric changes were recorded at 450 nm within 30 min, using an ELISA plate reader.

ELISA for IL-6 (D6050, R&D systems) was performed according to the manufacturer’s instructions. The antigen dilution series was extended for the ELISA to allow comparison to the UnFold and *in situ* PLA assays.

### Imaging

For all experimental conditions, at least three images were acquired. The microscope used was a Zeiss Imager Z2 controlled by the Zen 2 (blue edition) software. All images were taken with a 40×/1.4 oil objective and a Hamamatsu C11440 camera. The samples were excited by an HXP 120 V light source (50% light intensity) and imaged using filter cubes sets from Zeiss (number 49(#488049-9901-000), 38 HE (#489038-9901-000) and 43 HE (#489043-9901-000)) suitable for the fluorescence wavelengths of DAPI, FITC and Cy3. Exposure times were 40 ms for DAPI, 510 ms for FITC and 1.5 s for Cy3 during image acquisition.

### Image analysis

Image analysis and quantification were done using the CellProfiler software version 2.1.1, made available by the Broad Institute Imaging Platform^40^. A pipeline for signal quantification with slight modifications between the assays and experiments was created and built up by the following modules: *IdentifyPrimaryObjects*, *IdentifySecondaryObjects*, *EnhanceOrSuppressFeatures*, *MaskObjects* and *ExportToSpreadsheet*. First, *IdentifyPrimaryObjects* was used to identify any nucleus between 40 and 180 pixels and above a manually set threshold in the Hoechst stained images. Thereafter, seeding with the nucleus objects the *IdentifySecondaryObjects* module was used to expanding from the nucleus with 45 pixels or 80 pixels for HaCats and BJ hTert cells, respectively, to define the cells. The Cy3 images with the signals were first filtered to remove background larger than 12 pixels in diameter with a white tophat filter using the enhance speckles feature in the *EnhanceOrSuppressFeatures* module. The filtered images were thereafter run through another *IdentifyPrimaryObjects* module to detect signals, by setting a size limit to 1–12 pixels and a manual threshold of 0.025. Any signal outside of the defined cells was removed using the *MaskObjects* module to avoid any signals from cells not counted and therefore not included in the analysis. The data on the number of counted cells and masked signals per image were thereafter exported to a CSV file with the *ExportToSpreadsheet* module for calculations.

### Microtiter plate readout and analysis

The absorbance data were recorded using a Safire II microtiter plate reader, and the optical densities (ODs) at 450 nm, which is the optimal wavelength for measuring the products of the peroxidase reaction, were exported and further analyzed with Microsoft Excel software and ImageJ. The data were fitted using a four-parametric logistic regression model^41^ (1) in ImageJ (Curve Fitter > Rodbard). The assay system gives a sigmoidal curve and we used the 4 parametric logistic regression model because it provided the best curve fit. The model takes into account the minimum and maximum values, the point of inflection, and the slope of the curve. Thereafter, the model was used to estimate the concentrations for the limits of detection (LOD) and lower limits of quantification (LLOQ) by calculating the concentrations corresponding to the fitted curves at OD values corresponding to average background +3 standard deviations for LOD; and at average background +10 standard deviations for LLOQ.

1 $$O{D}_{value}=MaxOD+\frac{MinOD-MaxOD}{1+{(\frac{Concentration}{{\rm{Inflectionpoint}}})}^{CurveSlope}}$$

## Electronic supplementary material

Supplementry information
