# Supplementary material for: Improved efficiency of in situ protein analysis by proximity ligation using UnFold probes
Source: Sci Rep. 2018 Mar 29;8:5400. doi: 10.1038/s41598-018-23582-1 (PMC5876389; doi:10.1038/s41598-018-23582-1)
Supplement: Supplementary file 1 — Supplementry information [file 41598_2018_23582_MOESM1_ESM.pdf]

**Supplementary Information**

# Improved efficiency of *in situ* protein analysis by proximity ligation using UnFold probes

Axel Klaesson[1], Karin Grannas[1], Tonge Ebai[2], Johan Heldin[1], Björn Koos[3], Mattias Leino[1], Doroteya Raykova[1], Johan Oelrich[2], Linda Arngården[1], Ola Söderberg[1]*#, Ulf Landegren[2]*#

* Shared senior authors

[1] Department of Pharmaceutical Biosciences, Pharmaceutical Cell Biology, Uppsala University, Uppsala, Sweden.

[2] Department of Immunology, Genetics and Pathology, Science for Life Laboratory, Uppsala University, Uppsala, Sweden.

[3] Department of Systemic Cell Biology, Max Planck Institute of Molecular Physiology, Dortmund, Germany

#corresponding authors:

Ola Söderberg, Department of Pharmaceutical Biosciences, Uppsala University, Uppsala, Sweden, e-mail: ola.soderberg@farmbio.uu.se

Ulf Landegren, Department of Immunology, Genetics and Pathology, Science for Life Laboratory, Uppsala University, Uppsala, Sweden, e-mail: ulf.landegren@igp.uu.se

## Table of contents

**Page**

Supplementary figure S1 ……………………………………………………………………………………3

Supplementary figure S2 ……………………………………………………………………………………4

Supplementary figure S3 ……………………………………………………………………………………5

Supplementary figure S4 ……………………………………………………………………………………6

Supplementary figure S5 ……………………………………………………………………………………7

Supplementary figure S6 ……………………………………………………………………………………8

Supplementary figure S7 ……………………………………………………………………………………9

Supplementary figure S8 ……………………………………………………………………………………10

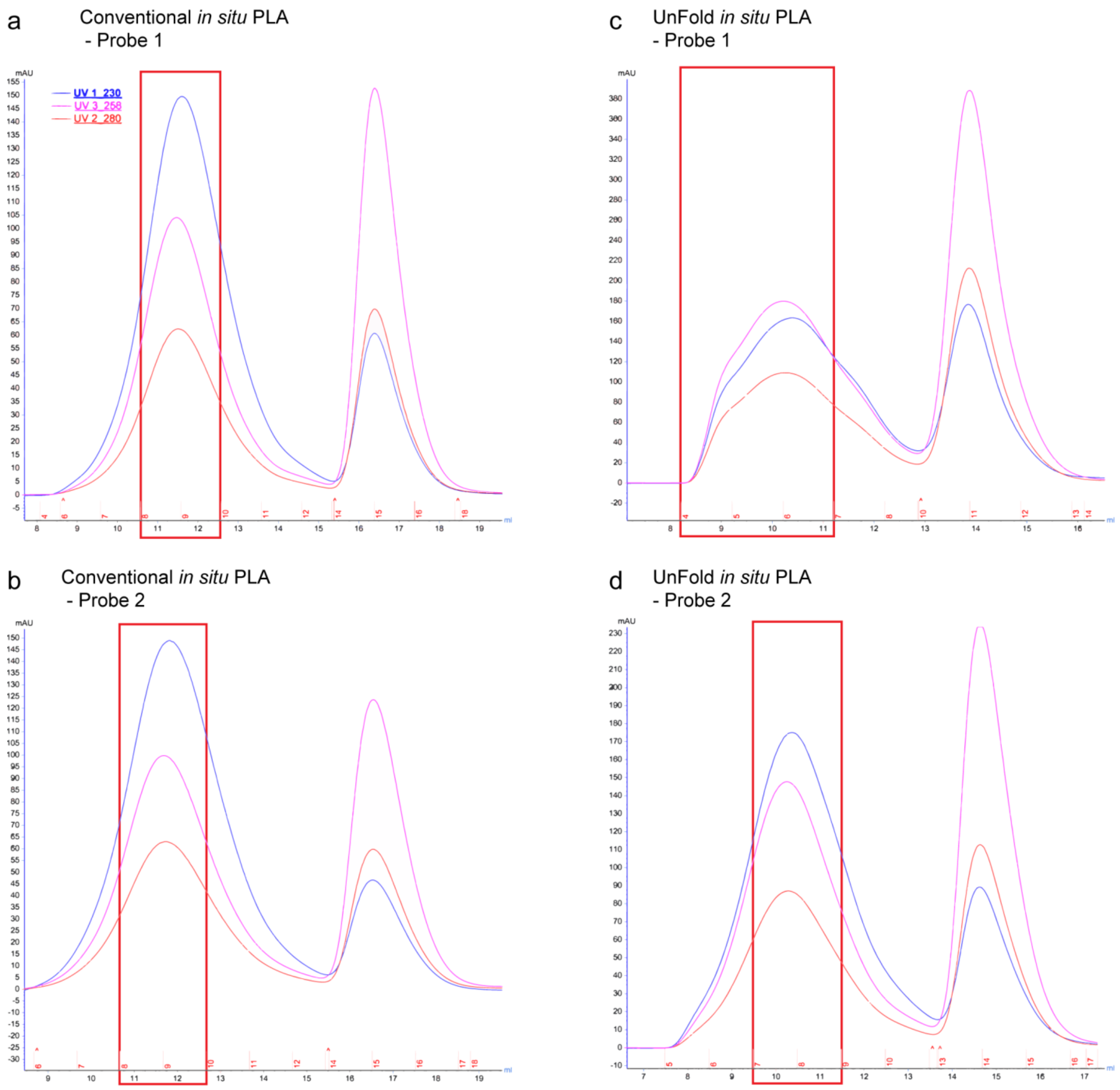


Supplementary Figure S1. **Chromatograms of the separation of conjugates.** After conjugation with their respective oligonucleotide each secondary antibody probe was purified by liquid chromatography on a Superdex 200 column. The conjugate flow-though was fractionated as annotated by the red numbers on the x-axis in the chromatogram: the fractions selected for *in situ* PLA have a red box surrounding them. The x-axis shows retention volume (ml) and the y-axis shows milli-arbitrary units (mAU) of absorption. The blue line shows absorption at 230 nm, the pink line shows absorption at 258 nm and the orange line shows absorption at 280 nm.

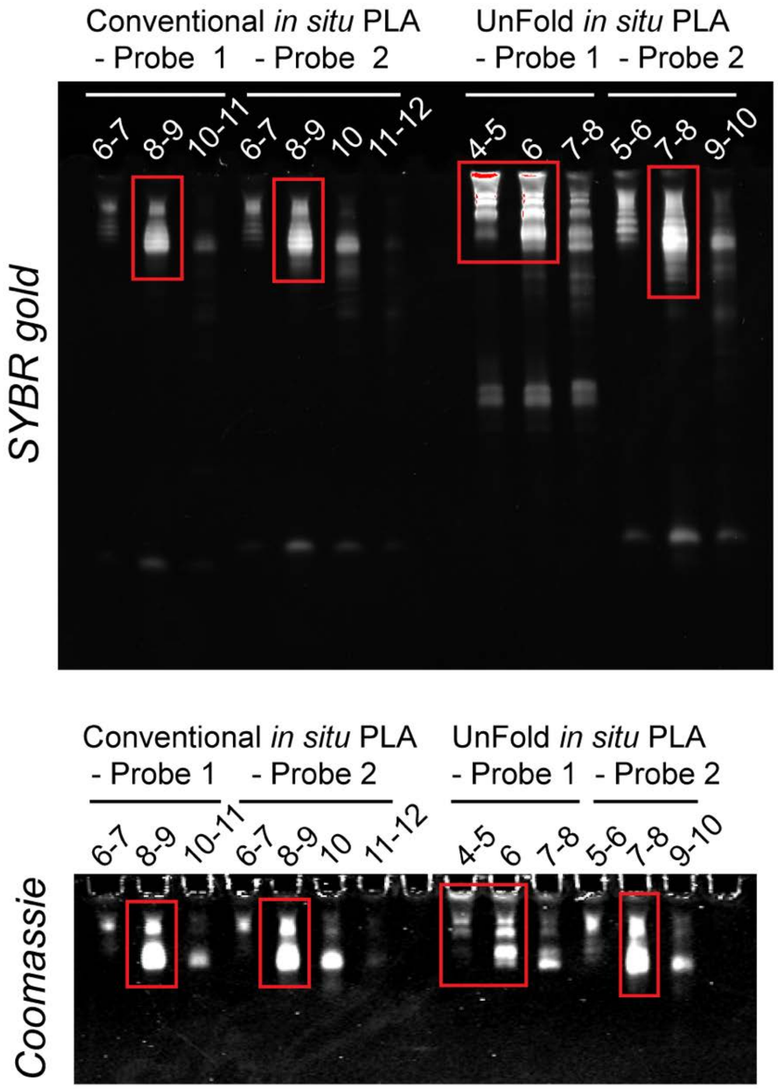


Supplementary Figure S2. **Gel electrophoresis of conjugate fractions stained with SYBR gold and Coomassie Blue.** Antibody-oligonucleotide conjugates were visualized by SYBR gold staining of DNA along with protein staining using Coomassie Blue. The red box show fractions selected for *in situ* PLA.

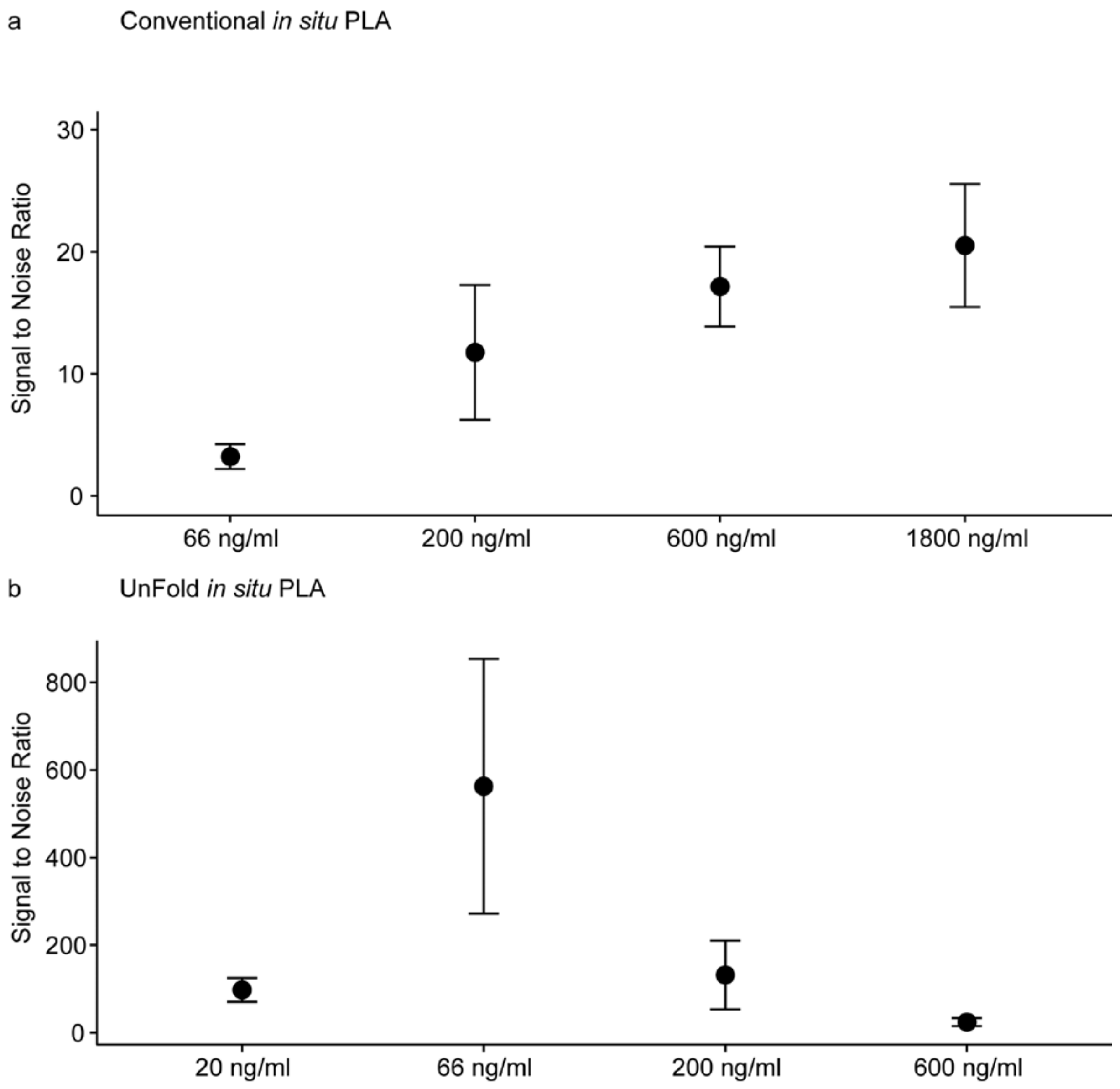


Supplementary Figure S3. **Detection of E-cadherin/β-catenin interactions in HaCat cells.** Conventional and UnFold *in situ* PLA probes were applied at four different concentrations. The signal-to-noise ratios were calculated as numbers of RCA products per cell from assays with primary antibodies present, divided by numbers of RCA products per cell from assays without primary antibodies (background). a) The conventional *in situ* PLA graph reflects results using probe concentrations from 66 ng/ml to 1800 ng/ml b) The UnFold graph represents experiments that included the concentrations 20 ng/ml to 600 ng/ml. Note the difference in scale between signals in panels a) and b). Experiments were performed three times. Error bars represent SEM.

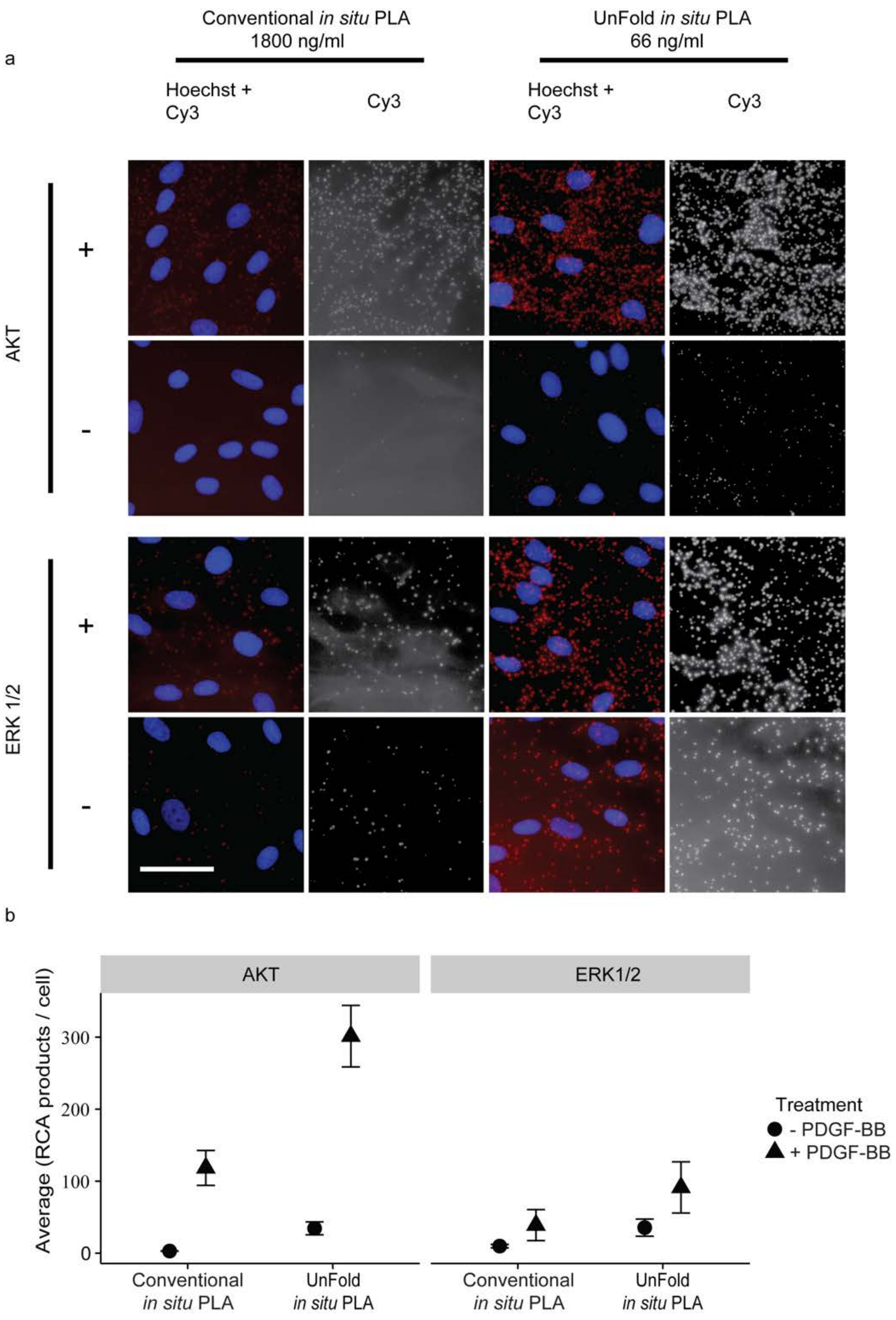


Supplementary Figure S4. **Visualization of phosphorylation of AKT and ERK1/2 signaling proteins in BJ hTert cells.** a) Serum-starved BJ hTert cells were treated with PDGF-BB for 15 min at 37°C (+) and compared to untreated cells (-). Both conventional *in situ* PLA and UnFold *in situ* PLA were used to visualize phosphorylation of AKT (anti-AKT and anti-pAKT) and ERK1/2(anti-ERK1/2 and anti-pERK1/2) in the cells. Nuclei were stained with Hoechst 33342 (blue) and RCA products labeled with Cy3 (red in merged image). Scale bar (white) = 50 µm. b) Signals were quantified and average RCA products per cells were plotted for comparison between conventional and UnFold *in situ* PLA. Experiments were conducted three times. Error bars represent SEM.

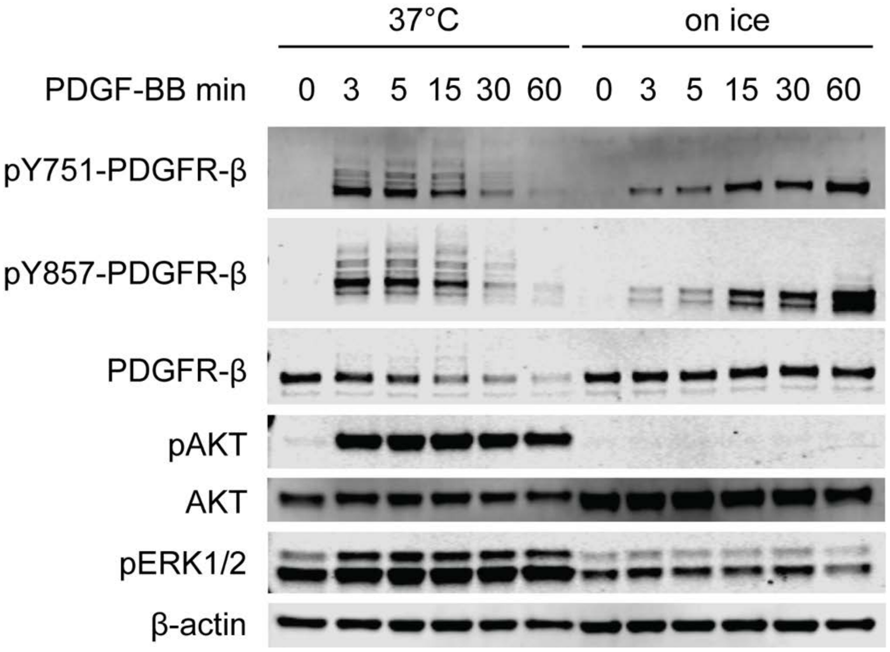


Supplementary Figure S5. **Western blot of stimulated BJ hTert cells.** BJ hTert cells where stimulated at 37°C or on ice for 0, 3, 5, 15, 30 and 60 min with 50 ng/ml PDGF-BB. When the BJ hTert cells where stimulated with PDGF-BB on ice a progressive increase of PDGFR-β phosphorylation was evident instead of the normal bell-shaped activation curve usually seen at 37°C, however, neither AKT or ERK was activated on ice above baseline phosphorylation, as compared to stimulation at 37°C, which showed a distinct increase of phosphorylated AKT and ERK1/2 upon receptor activation. Experiments were conducted three times. Whole blots in Supplementary Figure S6.

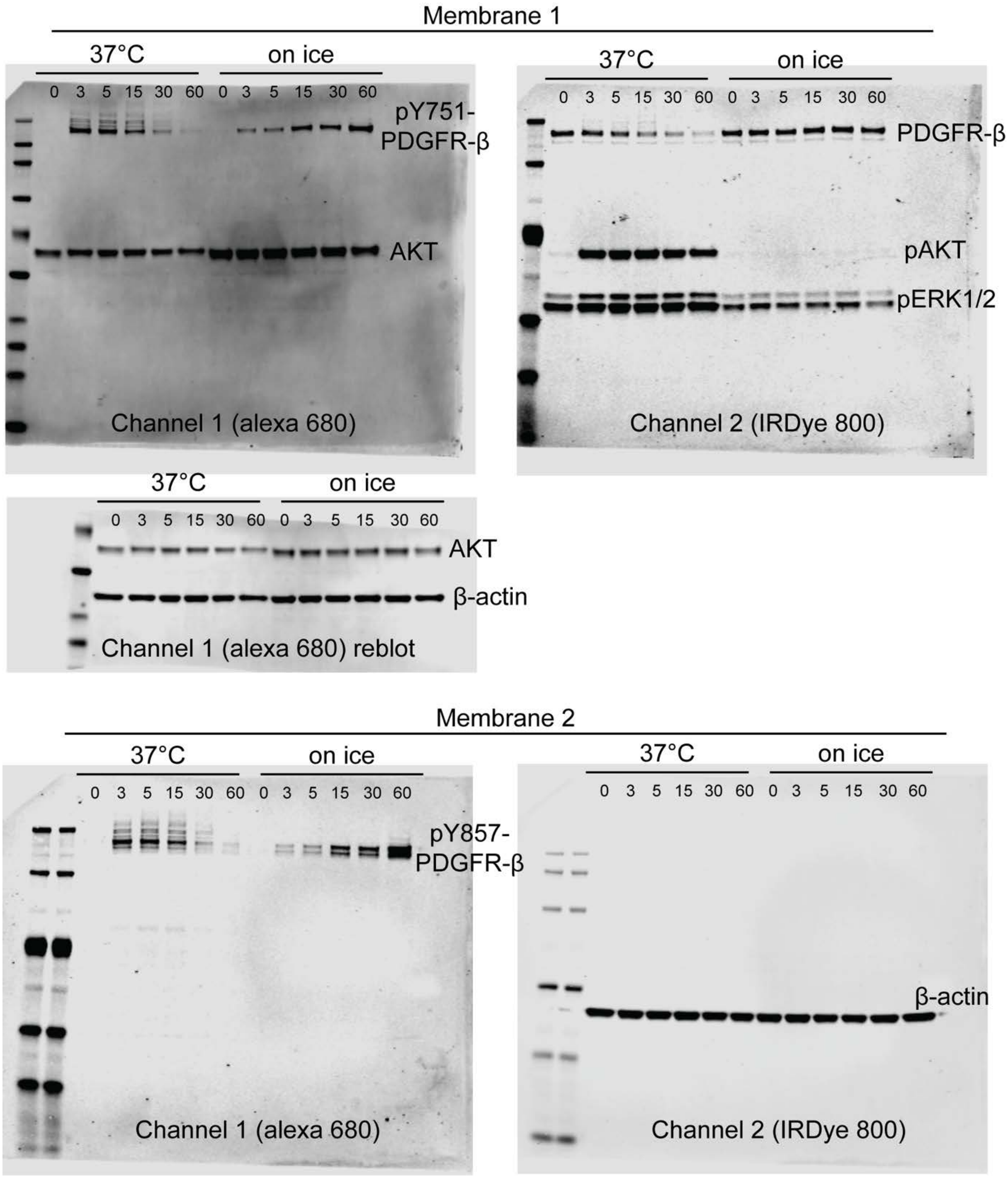


Supplementary Figure S6. **Western blot of stimulated BJ hTert cells (whole blots).** Whole blots from which the cropped blots in Supplementary Figure S6 were taken. Blots are marked with the primary antibody and the fluorophore-labeled secondary antibody with which it was stained.

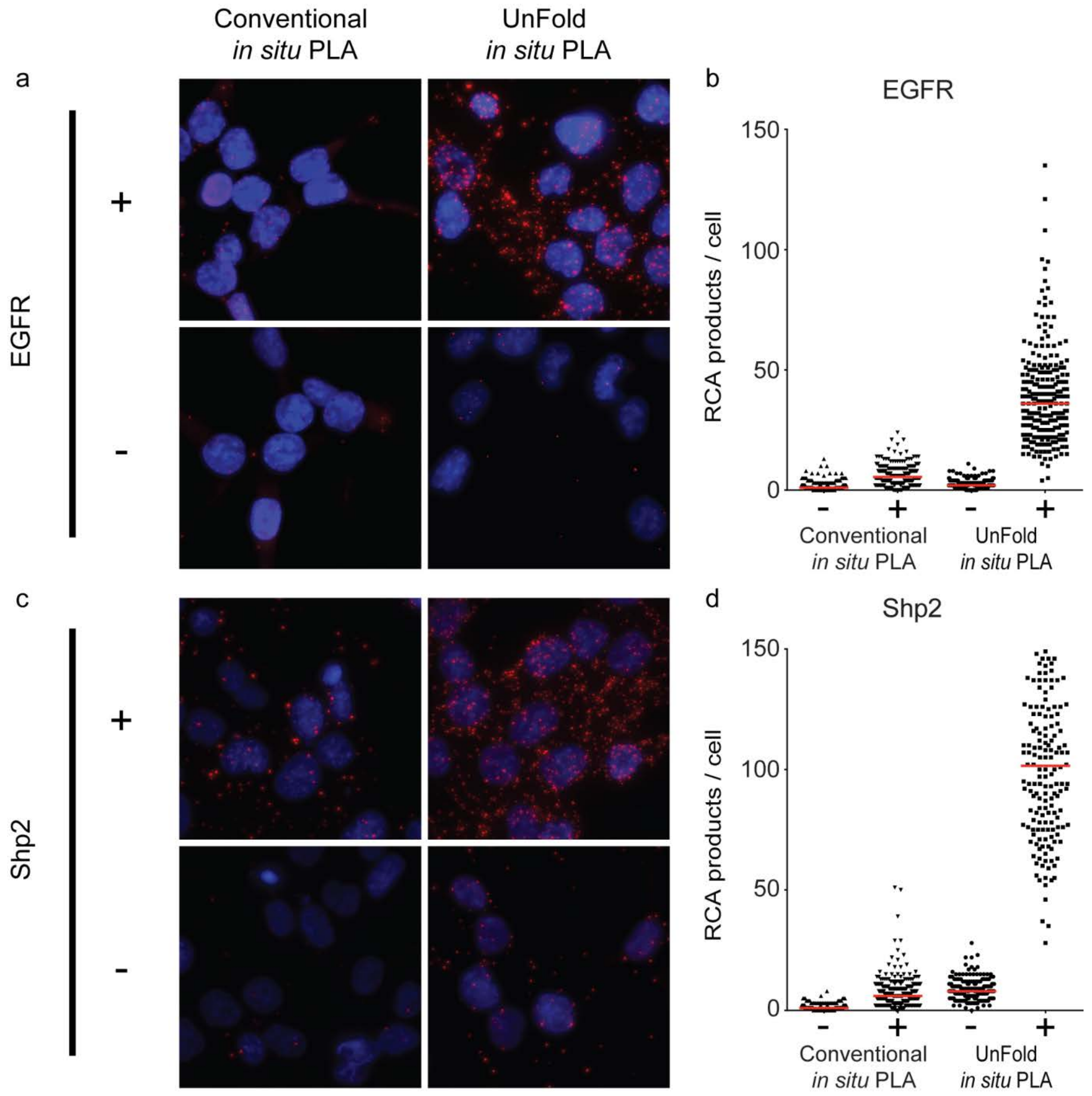


Supplementary Figure S7. **Visualization of phosphorylation of EGFR and Shp2 in HCT116 cells.** Serum-starved HCT116 cells were treated with EGF for 3 min at 37°C (+) and were compared to untreated cells (-). a) Conventional *in situ* PLA and UnFold *in situ* PLA were used to visualize phosphorylation of EGFR (anti-EGFR and anti-pEGFR) and b) RCA product in each individual cell was plotted. c) Shp2 phosphorylations (anti-Shp2 and anti-pShp2) was visulized using conventional *in situ* PLA and UnFold *in situ* PLA and d) results from the signal quantifications were plotted at a single cell level. Nuclei were stained with Hoechst 33342 (blue) and RCA products were labeled with Cy3 (red). The red lines in the two plots (b and d) represent the average number of RCA products per cell.

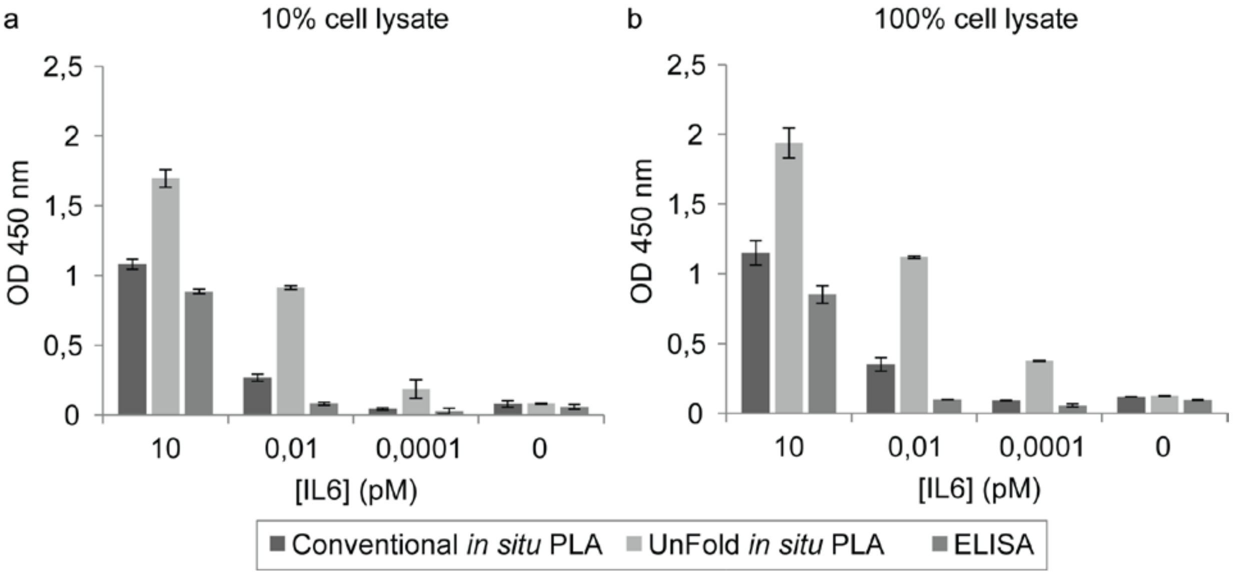


Supplementary Figure S8. **Measurement of recombinant IL-6 spiked in cell lysate.** 10, 0.01, and 0.0001 pM concentrations of IL6 was spiked in (a) 10% and (b) 100% cell lysate and captured in microtiter wells pre-coated with anti-human IL-6 antibodies. Captured IL-6 was detected via solid phase PLA using either conventional *in situ* PLA probes or probes with the UnFold design and the results were compared with those from a commercial sandwich ELISA. All reactions were monitored via peroxidase labeled reagents. Absorbance at 450 nm was read with a spectrophotometer after addition of the stop solution. Experiments were performed three times and one representative graph is showed. Error bars represent the standard deviation of duplicate reactions.
